# Mastering self-care: a qualitative exploration of the psychological and behavioral experiences of older heart failure patients living alone during hospital discharge preparation

**DOI:** 10.1186/s12912-026-04636-6

**Published:** 2026-04-24

**Authors:** Huiyue Zhou, Liqun Yao, Mengwei Shao, Menghan Zhang, Han Tang, Huibin Si, Anni Song, Mengru Zhang, Xiaowan Li, Ruofei Du, Na Zhou

**Affiliations:** 1 Nursing Department, Ninth People’s Hospital of Zhengzhou, Zhengzhou, Henan Province 450000 China; 2 Weifang Municipal Center for Disease Control and Prevention, Weifang, Shandong Province 261061 China; 3 School of Nursing and Rehabilitation, Shandong University, Jinan, Shandong Province 250012 China; 4 Anesthesiology Department, The First Affiliated Hospital of Zhengzhou University, Zhengzhou, Henan Province 450053 China; 5 School of Nursing, Wenzhou Medical University, Wenzhou, Zhejiang Province 325035 China; 6 The Second Ward of Cardiovascular Disease, Ninth People’s Hospital of Zhengzhou, Zhengzhou, Henan Province 450000 China; 7 Department of Lymphoma in Kaiyuan District, The First Affiliated Hospital of Henan, University of Science & Technology, Luoyang, Henan Province 471003 China

**Keywords:** Older people, Heart failure, Psychology, Behavior, Qualitative research

## Abstract

**Background:**

The transition from hospital to home presents significant psychological and practical challenges for older heart failure patients living alone (OHELP), who must navigate complex self-care needs within a socially isolated context. Although discharge preparation is a critical period for adapting to solitary living, the psychological and behavioral coping mechanisms of this vulnerable population remain underexplored.

**Objective:**

This study aimed to explore the psychological experiences during the pre-discharge period regarding self-care readiness, and to describe the behavioral strategies preferred by the OHELP.

**Methods:**

This descriptive qualitative study employed purposive sampling to recruit 26 participants from a geriatric hospital in Zhengzhou, China, between January and June 2025. Interview data were systematically organized and coded using NVivo 12, and reflexive thematic analysis was conducted through the research.

**Results:**

Four themes and twelve sub-themes emerged from the analysis: (a) anticipated psychological burden of living alone, (b) perceived deficits: needs vs. resources, (c) coping strategies for self-care and the defense of autonomy, (d) reconstructing one’s self-worth and meaning.

**Conclusions:**

The OHELP addressed anticipatory psychological burden during discharge preparation through adaptive strategies such as reestablishing daily routines and enhancing technological literacy, thereby reconstructing self-worth and maintaining autonomy.

**Clinical trial number:**

Not applicable.

## Introduction

China currently has approximately 280.4 million individuals aged 60 years and older, accounting for 19.8% of the total population [1]. Within this population, approximately 78% are living with at least one chronic disease [2]. Heart failure, a common and burdensome chronic condition among older adults, is associated with a poor prognosis and imposes considerable strain on the public health system due to its high readmission rates [3]. Given the aging population, the increasing number of older adults with heart failure necessitates effective long-term disease management, which critically depends on a seamless transition from the highly supportive hospital environment to a self-directed home setting [4]. During this transition period, particularly pre-discharge education and preparation, has been shown to significantly enhance patients’ self-care capabilities post-discharge [5]. Effective self-care of transitional care—defined as a series of autonomous decisions and behaviors undertaken by patients to maintain their life, health, and well-being—serves as a critical determinant of patient prognosis, quality of life, and readmission risk [6].

The transition from inpatient treatment to home care represents a particularly vulnerable period for patients with heart failure. Approximately 24.20% of these patients experience readmission within three months post-discharge, and 38.33% within six months [7]. At this stage, patients must navigate complex self-care tasks, including medication management, symptom monitoring, and lifestyle adjustments, while simultaneously addressing residual physical dysfunction and psychological burden [8]. These challenges are even more formidable for the OHELP. A meta-analysis indicates that, compared to patients living with others, those living alone exhibit significantly elevated risks of readmission and mortality, with hazard ratios of 1.78 at 30 days post-discharge and 1.24 at 90 days post-discharge [9]. Although the importance of discharge preparation is increasingly recognized, a critical gap remains in understanding how existing transitional care frameworks address the needs of vulnerable populations [10]. International expert consensus has highlighted the importance of multidisciplinary collaboration, caregiver involvement, and attention to the social determinants of health in optimizing the transition from hospital to home [10]; however, these recommendations remain largely generic. In China, research on transitional care is still fragmented, lacking unified implementation standards and offering little specific guidance for patients who live alone and lack immediate family support. This results in inadequacies in how patients perceive and navigate the care transition process [11, 12]. Specifically, two critical aspects warrant attention: First, there is an insufficient psychological risk assessment. While healthcare providers may recognize patients’ anxiety or depression, they often fail to identify these psychological responses as specific manifestations of the challenges associated with “living alone” [13]. Consequently, patients’ needs may be perceived as normal psychological responses rather than high-risk indicators that necessitate intervention. As a result, patients are frequently discharged with unresolved psychological burden [14]. Secondly, there is a lack of support for behavioral preparation. Standard discharge education often presumes the presence of a family member capable of providing support, particularly for older patients [15]. Discharge instructions typically emphasize “informing the family” rather than “empowering the patient.” However, for those living alone, healthcare providers may neglect to assist them in translating abstract medical advice (e.g., “low-salt diet”) into concrete, personalized action plans that can be executed independently. This oversight significantly increases the practical challenges faced by patients post-discharge, hindering their ability to effectively engage in self-care behaviors.

Inadequate psychological and behavioral support during the discharge preparation phase may lead to contrasting behavioral patterns among patients, ranging from excessive reliance—such as frequent emergency calls for minor discomfort—to treatment avoidance, including concealment of symptom deterioration due to fear of rehospitalization [16–18]. Leventhal’s Common-Sense Model of Self-Regulation (CSM) posits that when confronted with a health threat—such as the impending transition to solitary living with heart failure—individuals actively construct psychological experiences, drawing on their common-sense understanding derived from personal experience and available information [19]. This theoretical framework is particularly pertinent to investigating the experiences of patients living alone during the discharge preparation period. It suggests that a patient’s engagement with or avoidance of specific self-care behaviors is not merely a function of knowledge deficits but is fundamentally shaped by their idiosyncratic representations of living alone and the experiences these representations evoke [20]. For example, a patient who perceives the post-discharge situation as uncontrollable and experiences intense fear may adopt avoidance behaviors, whereas a patient with a stronger sense of personal control is more likely to actively seek information and formulate concrete self-care plans. Therefore, CSM can be utilized to investigate the psychological processing of the unique challenges encountered by individuals living alone, as well as how these processes subsequently influence the behavioral strategies developed by patients during this critical transition period. Addressing these knowledge gaps is crucial for fundamentally enhancing transitional care and achieving precision interventions [21]. Quantitative research approaches prove inadequate for capturing the dynamic psychological processes, such as anticipatory worries about solitary living, evolving fears, and fluctuations in self-efficacy, that patients experience [22]. In contrast, qualitative research offers an appropriate methodological pathway for investigating such complex, contextually embedded phenomena [23]. Through in-depth, context-based narrative communication, qualitative research can systematically illuminate the psychological experiences and behavioral logic of patients during transitional periods. This approach provides valuable guidance for the development of targeted, theory-based interventions aimed at supporting this vulnerable population [24].

Thus, this study aimed to employ a qualitative research approach to explore the psychological experiences during the pre-discharge period regarding self-care readiness, and to describe the behavioral strategies preferred by the OHELP.

## Methods

### Study design and setting

This study employed a qualitative descriptive design utilizing in-depth interviews to explore the psychological and behavioral experiences of the OHELP during the discharge preparation period. The reporting of this study adhered to the COREQ−32 checklist for qualitative research [25]. The research was conducted from January to June 2025 at the largest geriatric hospital in Zhengzhou, a central Chinese city. With 1,300 beds, two cardiology departments, and a dedicated cardiac rehabilitation center, the hospital can accommodate over 80 patients with heart failure simultaneously. Its advanced treatment capabilities and specialized nursing services ensure that patients receive the highest quality of care available in the region.

### Participants

Purposive sampling was employed to select participants. Inclusion criteria were as follows: (a) diagnosed with heart failure, (b) aged 60 years or older, (c) planned discharge within 24 to 72 h (refer to the supplementary material 1), (d) living alone after discharge (residing in a private residence without a cohabiting spouse, family member, or paid caregiver), and (e) able to communicate in Mandarin and willing to participate this study. Exclusion criteria were as follows: (a) patients with severe cognitive impairment or mental illness, as well as, (b) patients with other serious physical conditions that could potentially affect care outcomes.

All participants undergo three standard procedures prior to discharge: (1) a one-on-one discharge education session conducted by their responsible nurse, lasting at least 30 min and covering medication management, symptom monitoring, dietary management, activity guidelines, emergency response, and follow-up scheduling; (2) the provision of a written discharge rehabilitation handbook by the department; and (3) the scheduling of a post-discharge telephone follow-up appointment. In China, most medical expenses are covered by a combination of individual payments and government medical insurance, with some patients also possessing commercial health insurance. However, home-based care and in-person follow-up services require substantial out-of-pocket payments, which have consequently not been widely adopted [26]. Furthermore, due to constrained human resources and limited primary care infrastructure, the primary responsibility for post-discharge care largely falls on patients themselves.

### Data collection

First, participant selection and identification were conducted using purposive sampling combined with a maximum variation strategy to ensure significant diversity in terms of age, sex, educational level, living situation, and economic income, thereby capturing a broad range of perspectives [27]. The research team initially identified a pool of 42 eligible patients through the electronic medical record system based on the established inclusion and exclusion criteria. Preliminary communication regarding the study’s purpose, content, and requirements was undertaken with these individuals to assess their willingness to participate. Among them, three declined due to scheduling conflicts with routine medical follow-up appointments, four refused due to a lack of interest in the study, and two were excluded because of exacerbated physical discomfort. Ultimately, 33 participants confirmed their willingness to participate and met all study criteria.

Second, prior to the interviews, researchers provided each confirmed participant with a detailed and comprehensible explanation of the study’s specific content, which included the interview outline and discussion topics, its significance, potential risks (e.g., emotional distress arising from discussions about their illness experiences), and relevant requirements (e.g., the necessity to respond truthfully and the estimated interview duration). Participants were given at least 12 h to consider the information and ask questions of the researchers. After voluntarily agreeing to participate, they signed an informed consent form. Subsequently, researchers collected sociodemographic and clinical information from both the participants and their responsible nurses, including age, sex, educational level, area of residence, family situation, disease duration, New York Heart Association functional class, and ability to perform daily activities. This information was crucial for analyzing the relationships between participants’ psychological behaviors, life experiences, and their social background and clinical status, thereby laying the foundation for subsequent reflexive thematic analysis.

Third, researchers determined the timing and location of the interviews based on the participants’ preferences. Most interviews were conducted in the afternoon to avoid conflicts with morning therapeutic procedures, within a dedicated consultation room in the cardiology department. Each interview was face-to-face, involving one researcher and one participant, with an additional researcher present to facilitate communication, observe, and document non-verbal behaviors and emotional expressions (e.g., crying, laughing, hesitation, silence, or furrowed brows), ensuring the smooth progression of the interview process. All interviews were audio-recorded only after obtaining explicit consent from participants, with recording devices activated solely upon confirmed agreement. Each interview lasted between 60 and 70 min. The sample size was determined based on the principle of data saturation, with recruitment planned to cease once no new information emerged over three consecutive interviews, resulting in a minimum of 10 interviews conducted [28]. Although the research team initially identified 33 potential participants, data redundancy and information saturation were observed after conducting 23 interviews (each participant was interviewed only once, with no follow-up interviews). To confirm saturation, the research team conducted three additional interviews. After confirming that no new information emerged, it was concluded that no further interviews were necessary. The final sample consisted of 26 participants who completed the interviews.

#### Interview guideline

The semi-structured interview guideline utilized in this study was developed in alignment with the research objectives and the CSM. This guideline was evaluated by two clinical nursing specialists and underwent three pre-interview tests (Data from these pre-interviews were not included in the final analysis). The final interview guideline covered three key dimensions: representations, coping procedures, and appraisal (see Table 1).

**Table 1:** Semi-structured interview guide

CSM Dimension	Questions	Purpose
Representations	a) What emotions do you experience as you prepare to return home alone?, What specific concerns occupy your thoughts?	To explore participants’ emotional representations and cognitive concerns regarding their illness and impending discharge.
	c) Of all the challenges you’ve anticipated, which one do you perceive as being the most formidable?	To understand participants’ cognitive appraisal of illness consequences and personal control.
Coping Procedures	b) What strategies do you intend to implement to address potential challenges related to managing your health independently upon returning home (e.g., adhering to medication schedules, monitoring weight, regulating dietary intake)?	To identify the coping strategies participants plan to employ for self-management post-discharge.
	d) In moments when assistance is required, whom can you approach for support? What are your expectations regarding their help?	To explore participants’ coping resources and social support networks, reflecting the interpersonal dimension of coping.
Appraisal and Feedback	e) What particular area of guidance or support do you believe should be prioritized by the hospital prior to your departure?	To elicit participants’ evaluation of their discharge preparation needs and feedback on perceived gaps in support.
	f) Is there any other pertinent information that you consider significant but has not been addressed?	To provide an opportunity for participants to reflect on and appraise their overall preparation, allowing for unanticipated insights.

Note: **question C** appears under ***Representations*** as it probes perceptions of challenge severity, which relates to cognitive representations of consequences

#### Study team

The research team comprised five principal investigators and four research assistants. The principal investigators had extensive prior experience in qualitative research. The research assistants had received training focused on qualitative research methodology, including principles of qualitative inquiry, data collection techniques, and ethical considerations. To ensure consistency across the team, all members underwent additional study-specific training prior to data collection, which covered the interview protocol, communication strategies for engaging with participants, step-by-step procedures for conducting interviews, and precautions to maintain data integrity. The principal investigators were responsible for the research design, conducting interviews, data analysis, and drafting report. The research assistants supported the principal investigators by facilitating pre-interview communications, recording interview content, and organizing interview transcripts. A senior investigator oversaw and coordinated any ambiguous analytical findings to guarantee the accuracy and reliability of the data analysis.

### Data analysis

This study employed reflexive thematic analysis following Braun and Clarke’s six-phase approach [29], combining inductive, data-driven coding with theoretical sensitivity. While codes and initial themes were generated from participants’ narratives without a pre-existing template, the CSM served as a sensitizing concept—a source of questions and foci that directed attention to aspects of the data relevant to self-regulation processes. This method was well-suited to exploring the subjective experiences and meaning-making processes of participants—central to the study’s aim of understanding the psychological and behavioral experiences of this vulnerable population during the discharge transition [30].

The main procedures included: (1) Familiarization with the data. Two analysts independently read and re-read the interview transcripts multiple times to gain a deep understanding of the content. Initial thoughts and observations were recorded in memos. (2) Generating initial codes. Working independently, both analysts systematically coded the entire dataset line-by-line, identifying meaningful units of text relevant to the research questions. No pre-established categorization framework was used; coding was inductive and data-driven, allowing codes to emerge from the participants’ narratives. However, during the coding process, both analysts remained attentive to aspects of the data that resonated with the theoretical constructs of the CSM, such as psychological and cognitive representations, while remaining open to unexpected patterns. (3) Searching for themes. After completing independent coding, each analyst collated their initial codes into potential themes by grouping related codes together. This involved sorting and combining codes to form broader patterns of meaning. (4) Reviewing themes. The two analysts met to compare their independently developed themes. They discussed the similarities and differences in their conceptualizations, referring back to the coded data and full transcripts to ensure themes accurately represented the data. Disagreements were resolved through discussion; when consensus could not be reached, a third senior investigator was consulted to review the data and facilitate a final decision. Themes were then refined, merged, or discarded through this collaborative process. (5) Defining and naming themes. Once a final set of themes was agreed upon, both analysts collaboratively developed a detailed analysis of each theme, defining its scope, core content, and sub-themes. Clear, descriptive names were assigned to each theme. (6) Writing the report. The final themes were written up as part of the research findings, with illustrative quotes selected to represent each theme.

### Rigour

To ensure the rigor and credibility of the interview data, this study employed triangulation and member-checking strategies. Triangulation involved cross-validating participants’ responses through the analysis of electronic medical records, nursing documentation, and discharge readiness procedures. Due to challenges in maintaining effective communication with post-discharge participants via email—particularly as some older and low-literacy participants lacked proficiency in using email—the research team selectively invited certain participants for member checks. Transcriptions were promptly completed after interviews to allow for review prior to discharge. Preliminary thematic findings were communicated via telephone, and subsequent refinements to the research outcomes were made based on the feedback received. Furthermore, to ensure the reproducibility of the research, all procedures—including interview guides, data transcription, and analysis steps—were meticulously documented. Regular team discussions and reflective practices, such as recording potential biases, were conducted to enhance the credibility and reliability of the research findings.

## Results

### Characteristics of participants

A total of 26 participants (7 females and 19 males) were included in the final analysis, at which point data saturation was achieved—defined as three consecutive interviews yielding no new themes. All participants lived alone prior to admission and were scheduled to return to solitary living upon discharge. Detailed demographic characteristics were presented in Table 2.

**Table 2:** Demographic characteristics of participants

Category	Subcategory	*N* = 26
Gender		
	Female	7
	Male	19
Age (years)		
	60 ~ 69	7
	70 ~ 79	10
	80 ~ 89	9
Educational level		
	Primary school or less	10
	Secondary school	13
	College degree or above	3
Resident area		
	Rural area	5
	City	21
Duration of disease (year)		
	<5	7
	5 ~ 10	13
	>10	6

### Description of themes and subthemes

Thematic analysis of the 26 interview transcripts revealed four major themes capturing the psychological and behavioral experiences of participants during the discharge preparation period. These themes were developed from data collected up to the point of saturation, with the final three interviews serving to confirm rather than extend the thematic structure presented below. Grounded in the CSM theory, these themes illustrated the parallel processing of psychological experiences during the transition to independent living, the coping strategies employed, and the continuous evaluation and meaning-making processes that shaped participants’ self-regulation. As shown in Table 3, the four themes identified were: (a) anticipated psychological burden of living alone, (b) perceived deficits: needs vs. resources, (c) coping strategies for self-care and the defense of autonomy, and (d) reconstructing one’s self-worth and meaning.

**Table 3:** Themes and sub-themes

Conceptual components of the CSM	Theme	Sub-theme
Representations (Associated cognitive and psychological experiences)	Anticipated Psychological Burden of Living Alone	The core challenge: fear of encountering a medical crisis
		Anticipatory loneliness and forced independence
		Heightened alertness accompanied by emotional fatigue
	Perceived Deficits: Needs vs. Resources	Prioritized support needs
		Perceived resource availability
		Critical gaps
Coping procedures (Behavioral responses)	Coping Strategies for Self-Care and the Defense of Autonomy	Problem-focused coping: reconstructing life routines
		Experience-based coping: developing an empirical knowledge system
		Psychological-focused coping: balancing strategic dependence
		Technical literacy as a coping resource
Appraisal and feedback (Dynamic appraisal and feedback effects)	Reconstructing One’s Self-Worth and Meaning	Weaving a web of meaning
		Coexisting with chronic illnesses

## Themes 1: anticipated psychological burden of living alone

### Sub-theme 1.1: the core challenge: fear of encountering a medical crisis

When asked about the most severe challenges they anticipated facing, the participants unanimously identified the potential for medical crises—such as falls, breathing difficulties, or heart attacks—occurring without anyone nearby to notice, respond, or call for help. These scenarios predominantly occupied their thoughts during the discharge preparation phase. They were not merely abstract concerns; rather, they evolved into a persistent underlying fear.

*In the hospital*,* nurses conduct nightly rounds; however*,* at home*,* it is just me and the ceiling. Occasionally*,* when I sense my heart racing*,* I swiftly sit up*,* fearing the prospect of falling asleep and not waking up again. (P2)*

*The reason for this hospitalization was a fall at home. Consequently*,* I now experience anxiety at the thought of returning home*,* fearing that I may fall again. (P7)*

*The biggest worry? No one to call 120*,* no one to help me to the bed*,* no one to even know I’m in trouble. (P14)*

### Sub-theme 1.2: anticipatory loneliness and forced independence

Beyond specific fears about emergencies, participants described a deeper, more existential layer of psychological experience: anticipatory loneliness. This was not simply the absence of company, but the profound awareness that they would bear full responsibility for their own survival—that in moments of crisis or need, there would be no one else to share the burden.

*Although life in the hospital can feel inconvenient*,* I have fellow patients to converse with; in contrast*,* at home*,* I am alone in an empty space.(P7)*

*What options do I have? My spouse is no longer present*,* and my children have established their own families. I do not wish to become a burden to them. After all these years*,* I have grown accustomed to being self-reliant… (sigh). (P10)*

*It’s not that I’m afraid all the time. It’s more that I deeply know*,* when something important happens*,* I’ll have no one to rely on. That feeling weighs on your heart like a mountain. (P22)*

### Sub-theme 1.3: heightened alertness accompanied by emotional fatigue

The fear of safety risks associated with living alone compelled participants to engage in continuous self-monitoring, resulting in a prolonged state of hypervigilance that ultimately culminated in psychological exhaustion and feelings of depletion.

*Previously*,* when I was at home*,* I consistently monitored my heart rate. The moment it increased even slightly*,* I would become anxious. (P10)*

*It is essential to document weight*,* fluid intake*,* and fluid output. There exists a persistent tension in my mind*,* constantly preoccupied with concerns about swelling. (P13)*

## Themes 2: perceived deficits: needs vs. resources

### Sub-theme 2.1 prioritized support needs

When directly asked which services hospitals should prioritize providing before discharge, participants clearly identified three specific categories of support.

#### Practical guidance for daily solo living

Participants expressed dissatisfaction with generic discharge instructions that assumed the presence of caregivers. They required practical, step-by-step guidelines for performing self-care tasks independently.

*What I need are practical tips—how to cook for one person while following a low-salt diet*,* how to go grocery shopping*,* how to plan my meals. Doctors assume that someone is cooking for me. (P11)*

*And when I feel dizzy*,* how am I supposed to measure my blood pressure on my own? They just tell you what to do*,* but they don’t tell you how to do it when you’re alone. (P19)*

#### Clarifying the pathway to post-discharge services

Participants not only sought information about existing services, but also expected clear and simplified guidance on how to access these services effectively.

*I heard that home doctor services are available for application*,* but the registration process is complicated and confusing. I wish you could help get everything sorted—that would be more useful than all the brochures. (P18)*

#### Continuity of care support

Participants expressed the need for ongoing support that extends beyond a singular discharge education session. They hoped to maintain a continuous connection within the healthcare system.

*I prefer to have regular phone conversations with you to discuss some health management issues. (P24)*

### Sub-theme 2.2: perceived resource availability

#### Informal support: present but strategically utilized

While informal support networks rooted in familial ties and neighborhood relationships were present, their availability was intermittent and contingent upon circumstances beyond participants’ control—such as children living in distant cities with limited capacity for frequent involvement, or neighbors whose own commitments could preclude sustained attention. Participants contended that these resources ought to be utilized judiciously, exclusively in genuine emergencies, and should not be integrated into everyday life.

*My child is in Shanghai*,* overwhelmed with work pressure. I would rather manage these minor issues myself—what is the point of burdening them? (P21)*

*The neighbors are amicable individuals; however*,* they have their own lives to lead. It is not appropriate to disturb them too frequently. (P9)*

#### Formal support: distant and difficult to access

Given the limited availability and inconvenience of formal support resources, such as accessible community services and family doctors, participants perceived these resources as distant, abstract, and associated with negative expectations.

*Community hospitals? They are often perceived merely as facilities for routine services such as blood pressure checks or the prescription of over-the-counter medications. Given my heart condition*,* I question their capacity to adequately address my medical needs. (P11)*

### Sub-theme 2.3: critical gaps

Three significant gaps emerged between the support needs anticipated by participants and the resources available to them. First, participants generally lacked the ability to live independently and manage their health during periods of illness. Second, while medical services were technically accessible, participants faced challenges in navigating complex procedures and unclear pathways to access these services. Third, most participants tended to lose contact with medical staff following discharge, leading to interruptions in the continuity of their support.

*But the nurses would tell me I need to reduce my salt intake*,* but they wouldn’t teach me how to do it on my own. (P19)*

*The doctor said I could seek help from the community health center. But where is it? Do I need to bring some documents? It’s all too complicated. I gave up. (P22)*

*In the hospital*,* someone came to see me every day. But the moment I walked out of the hospital gates*,* there was nothing. No calls*,* no messages. It’s as if they’ve already forgotten about me. (P24)*

## Themes 3: coping strategies for self-care and the defense of autonomy

Participants developed various coping strategies adapted to the demands of living alone, aiming to both manage their condition and maintain autonomy.

### Sub-theme 3.1: problem-focused coping: reconstructing life routines

In the community support model, problem-oriented coping strategies are designed to directly address the threats at hand. For participants living alone, this entails the translation of abstract medical advice into specific, actionable plans.

*I developed a five-color grid to systematically organize my daily medications and set alarm reminders for their consumption. Each morning*,* immediately after waking*,* I record my weight and heart rate; this routine is as habitual as clocking in for work. (P8)*

*I cook three days’ worth of meals at once and portion them out. When I want to eat*,* I just heat up one portion—it’s convenient and ensures I always have food ready. This is what we older living alone call ‘standard operating procedure’. (P18)*

### Sub-theme 3.2: experience-based coping: developing an empirical knowledge system

In the absence of immediate at-home support to validate their concerns or guide their decision-making, participants learned to rely on their own observations and experiences to cope. They developed a response strategy that combined cognitive representations of illness with experiential learning.

*Although the doctor’s orders did not specify certain details*,* I still explored my physical limits in daily life. For instance*,* consuming one apple would not burden my appetite*,* whereas eating two would cause stomach discomfort. (P11)*

*I am fully aware of how temperature fluctuations impact my well-being. A mere 5°C drop in ambient temperature can immediately halt my outdoor activities. Previous experiences have demonstrated that venturing outside under such conditions would trigger my illness. (P20)*

### Sub-theme 3.3: psychological-focused coping: balancing strategic dependence

The CSM theory posits that individuals adopt strategies to cope with psychological stress related to health threats. For participants, the absence of immediate in-home support increased psychological pressures, such as tension and anxiety, while also compelling them to proactively explore self-care techniques and methods for seeking assistance. In identifying their support needs, they simultaneously maintained autonomy in their daily lives.

*Living alone has taught me that dependence doesn’t have to be one-way—it can be a kind of mutual exchange. I often collect packages for my neighbors*,* and in return*,* they help me with heavy items. (P13)*

*While I decline to have my children visit me daily*,* I do encourage them to call often. This arrangement ensures that they are aware of my well-being*,* and it alleviates my concerns about feeling abandoned. (P23)*

### Sub-theme 3.4: technical literacy as a coping resource

Participants endeavored to learn and utilize new technologies—such as internet-based health monitoring platforms, AI-assisted applications, and automated reminder systems (e.g., automated voice call reminders, or notification functions on wearable devices such as smartwatches)—to enhance their self-management capabilities. In this process, the value of technology transcended its practical utility as an external tool, evolving into a form of empowerment and affirmation of patients’ intrinsic abilities.

*My current smartwatch and mobile phone enable simultaneous monitoring of my heart rate. In the event of an emergency*,* a distress signal will be dispatched to inform my daughter. It always watch over me. (P4)*

*Although it’s difficult to learn*,* I need some smart devices to serve as a “home-based health assistant”*,* such as those that can monitor exercise*,* track nutrition*,* etc. These would be very useful. (P25)*

## Themes 4: reconstructing one’s self-worth and meaning

In the prolonged battle against illness and loneliness, some participants exhibited profound reflection and adaptability. By finding new meaning in solitary living and embracing the concept of coexisting with illness, they discovered their own ‘fulcrum’ for resistance.

### Sub-theme 4.1: weaving a web of meaning

To sustain their perseverance, participants actively reconstruct the narratives of their lives, seeking or even creating new values and purposes amidst adversity.

*This heart is akin to an old companion. Although it occasionally exhibits erratic behavior*,* it is essential to accommodate and soothe it. Adhering to a timely medication schedule and ensuring adequate rest are fundamental strategies for coexisting harmoniously with this condition. It does not define my entire being; rather*,* it constitutes merely a segment of my life. (P22)*

*Yourself have to find purpose in the small things—the daily routines become your reason to get up in the morning. (P24)*

*I approach self-care as a new vocation. I derive significant satisfaction from effectively managing my physical health indicators and organizing my daily routine. This endeavor serves as part of a new career. (P26)*

### Sub-theme 4.2: coexisting with chronic illnesses

Participants living alone had developed a concept of ‘companionship with illness,’ wherein self-care was internalized as a positive life attitude and regarded as an essential daily ritual.

*I am determined to live well enough to witness my granddaughter’s journey through college. This aspiration occupies my thoughts at present. Consequently*,* regardless of the challenges I may face*,* I will diligently take my medication and prioritize my health. (P3)*

*Though I find myself alone*,* I strive to infuse richness into my life experiences. Each day*,* I engage in listening to operas*,* basking in sunlight on the balcony*,* and observing children playing below me. The act of living itself holds intrinsic meaning. (P17)*

## Discussion

This qualitative study provided an in-depth analysis of the psychological and behavioral experiences of the OHELP during their preparation for hospital discharge. Grounded in the CSM theory, the study identified participants’ psychological and cognitive representations, coping strategies, evaluations, and feedback [31]. The findings revealed that participants’ pre-discharge representations—such as anticipatory psychological burden and perceived resource deficits—influenced the adaptive strategies they employed to address challenges, ultimately facilitating the process of self-value reconfiguration. This study offered critical insights for improving transitional care.

This study revealed that the OHELP experienced anticipatory psychological burdens prior to discharge, primarily encompassing fear of medical crises, anticipated loneliness, and vigilance fatigue. Previous research had confirmed that heart failure patients often harbor fears regarding disease deterioration [32]; however, the findings of this study indicate that for those OHELP, such fears frequently escalated into catastrophic thinking—where loneliness transformed normal bodily perceptions into sources of distress. This pattern may be attributed to the abrupt transition from a structured, supervised hospital environment to complete self-management at home, which exacerbates the perceived burden of independent self-care [33]. Returning to a quiet and isolated home environment might undermine patients’ confidence in managing their condition, diminish self-efficacy, and heighten feelings of helplessness and anxiety when coping with symptom fluctuations [34, 35]. Within the cultural context of filial piety in China, the absence of family or social support for those living alone not only exacerbated the objective lack of resources but also engendered a subjective sense of abandonment [36]. This dual burden impeded treatment adherence and accelerated the deterioration of cardiovascular function [37], thereby creating a vulnerability cycle: pre-discharge psychological burden diminished patients’ perceived availability of support resources, which in turn reinforced the initial psychological burden. Therefore, transitional care programs should have specifically addressed the psychological burden of the OHELP, aiming to mitigate catastrophic ideation and to enhance their sense of security and control. Notably, in contrast to Western contexts with well-established community care systems, home-based care remains the predominant form of older care in China [38]. Consequently, establishing reliable family support networks and personalized follow-up services constitutes the primary approach to optimizing transitional care for the OHELP.

This study revealed a significant mismatch between the support resource needs and actual access experienced by the OHELP. This mismatch was specifically manifested in the lack of practical guidance for independent daily living, the absence of clear pathways to access existing services, and the discontinuity of professional support following discharge. The CSM posited that individuals’ cognitive representations of a health threat influenced their appraisal of available coping resources [19]. These findings extended this theoretical tenet by demonstrating that for OHELP, the cognitive representation of living alone triggered a heightened sensitivity to resource inadequacy. Although discharge education for patients with heart failure has continuously improved [39], this study confirmed that such generic discharge education failed to address the unique challenges posed by living alone. The OHELPs’ expressed need for tailored, actionable, step-by-step guidance specific to their solitary living situation underscored the significant gap between standardized medical advice and the practical realities of managing chronic conditions in isolation [40]. This reflected a broader issue within the Chinese healthcare system, wherein discharge planning remained hospital-centered rather than community-integrated, resulting in discharge education that was overly theoretical and disconnected from patients’ actual life circumstances [41]. Notably, OHELP intentionally reserved informal support from family members or neighbors as a means of coping with genuine emergencies to avoid becoming a long-term burden—a phenomenon deeply rooted in traditional Chinese cultural values of reciprocity and social dignity [42]. This “strategic restraint” meant that available support was perceived as unstable rather than reliable, thereby further reinforcing their psychological burden. In contrast, the perceived distance to accessible formal support primarily stemmed from inadequate resource allocation. The current community- and primary-level healthcare system in China was characterized by significant regional disparities [43]; community hospitals, particularly those in economically underdeveloped areas, were often unable to provide timely and effective medical assistance, while the development of family physician services lagged even further [44]. This situation compelled OHELP to maintain low expectations regarding the availability of formal support following discharge. Additionally, the OHELP described the post-discharge period as a sudden “cliff edge,” where the daily attention received during hospitalization abruptly disappeared outside the hospital, highlighting the lack of transitional care mechanisms. These findings suggested that current discharge practices were predicated on assumptions regarding caregiver availability that did not accommodate the OHELP. Therefore, for the OHELP independently, transitional care models needed to take into account their family contexts and bridge community health resources according to rehabilitation needs upon discharge. Furthermore, developing a structured follow-up plan was essential to ensure that continuous support could be provided at home.

The CSM theory posited that individuals were not passive recipients of illness; rather, they actively sought explanations and coping mechanisms based on their commonsense understanding of their health conditions [19]. In response to the representations of anticipatory psychological burden and perceived lack of support resources exposed by living alone, OHELP primarily adopted strategies of reestablishing life routines, constructing experiential knowledge systems, balancing strategic dependency, and utilizing technological literacy to maintain self-care and preserve their autonomy. Reestablishing life routines served as a foundation for establishing a sense of control. This served as a behavioral response to the cognitive representation of “chaos” and “unpredictability”—by transforming vague medical advice, such as “monitor symptoms,” into concrete, repeatable tasks (e.g., recording vital signs in the morning) [45]. This behavior effectively transformed uncontrollable health threats into manageable tasks, alleviating anxiety while creating a predictable and psychologically safe space for OHELP themselves [46]. Constructing experiential knowledge systems illustrated how OHELP transitioned from passive reception of medical information to active knowledge integration—a process reflecting individual agency in problem-solving. By integrating objective medical guidance (e.g., the principles of sodium restriction) with subjective bodily sensations (including the degree of edema experienced after consuming certain foods), OHELP were able to construct a personalized and contextualized system of “practical knowledge.” This not only enhanced self-efficacy but also promoted autonomy. Therefore, nursing practice should actively encourage and respect this experiential wisdom of the OHELP, considering it a vital resource for optimizing individualized care plans [47]. Balancing strategic dependency reflected a culturally nuanced coping strategy. The concept of “relational autonomy” embedded in this behavior challenged the assumption that care dependency necessarily undermines autonomy [48]. Within the Chinese cultural context, where collectivist values emphasize reciprocity and “not burdening others” [49], this “mutual assistance” model enabled individuals to exercise control over when, from whom, and how they sought help—a selective approach that preserved dignity while ensuring safety.

This study emphasized ‘technological literacy’ as a previously underrecognized pathway for positive adaptation. Unlike traditional forms of social support or hobbies highlighted in earlier studies, the mastery of technology—such as the use of smart health devices—offered unique empowerment experiences for the OHELP. At an instrumental level, technology acted as a direct external extension that enhanced the safety and convenience of self-monitoring, thereby strengthening individuals’ sense of control over their health status [50]. Psychologically, the successful learning and application of new technologies served as a powerful means for the OHELP to showcase their capabilities, and maintain connections with modern society [51]. However, age-related cognitive decline, financial constraints, and fear of learning new technologies might exclude some of the OHELP from digital health services [52]. Therefore, it was essential to develop age-friendly smart devices, simplify user interfaces, and provide patient-centered, repetitive technical training support to assist the OHELP in overcoming practical challenges associated with technology use. Governments should integrate digital health literacy into the support system for chronic disease management among the older, alleviating financial barriers to smart health devices through subsidies or free distribution [53]. For the OHELP who could not access or struggle to master new technologies, traditional non-technological self-monitoring tools should still be employed, while strengthening non-technological social support networks, such as regular telephone follow-ups, the establishment of mutual aid groups, and volunteer home visits [52]. It was crucial to emphasize that the introduction of technology should not replace interpersonal care, but rather serve as a supplementary tool to enhance the self-efficacy of the OHELP. Nursing practice should provide personalized and diverse support plans based on the OHELPs’ specific circumstances and preferences, rather than viewing technological applications as the sole ‘advanced’ approach. Regardless of whether technological tools are utilized, the core objective remained to enhance the OHELPs’ sense of control, security, and self-efficacy, assisting them in rebuilding meaning and dignity in life while coexisting with illness.

This study found that OHELP who successfully implemented coping strategies derived a sense of achievement and meaning from effective self-care, which alleviated their initial psychological burden and reinforced positive self-perception. This finding corroborated previous research suggesting that self-care served as a source of meaning [53], while also indicating that meaning reconstruction served a compensatory function. In the absence of immediate social support from cohabiting family members, patients had to cultivate intrinsic motivational resources to sustain the demanding work of self-care. Nursing interventions were not to focus solely on clinical outcomes and compliance; instead, they were to actively explore and reinforce patients’ sources of meaning, assisting them in articulating personal goals that extended beyond mere survival and connecting daily self-care tasks with these broader life narratives. Interventions that facilitated meaning-making—such as narrative-based approaches and value-congruent goal setting—were suggested to enhance psychological resilience and foster sustained engagement in self-care among the OHELP [44].

## Implications

Based on the CSM, this study identified that OHELP experienced a dynamic, iterative process involving the interplay of representations, coping procedures, appraisal and feedback during the discharge transition. Anticipatory psychological burden (cognitive/emotional representations) influenced perceived resource deficits (appraisal of coping resources), which in turn motivated the formation of coping strategies (coping procedures). Successful coping fed back into positive self-value reconfiguration (appraisal), which subsequently moderated the initial psychological representations. The findings of this study have significant implications for optimizing discharge preparation and long-term management of the OHELP. First, it is essential to incorporate targeted psychological interventions into discharge planning [54]. Healthcare providers should proactively assess patients’ psychological burden, acknowledge and validate their fears and feelings of loneliness, and offer psychological counseling or stress management techniques to alleviate catastrophic thoughts and emotional fatigue [55]. Second, personalized self-care education is crucial; discharge education should extend beyond generic guidelines to address the practical needs of solitary living. This includes teaching patients to develop individualized medication and dietary plans, sharing experiences of ‘strategic dependence,’ and assisting them in balancing autonomy and support. Third, it is vital to strengthen and integrate support systems. Clinicians and nurses should collaborate with community health service institutions to enhance the accessibility and professional capacity of formal support, such as establishing home-visit service platforms that connect patients with their families [56]. Additionally, healthcare professionals can facilitate the creation of informal support networks and encourage patients to proactively seek help by alleviating their fears of becoming a burden. Fourth, promoting the application of digital health technologies is essential. Medical institutions can provide training for older patients on using user-friendly smart devices, enabling them to utilize technology for health monitoring and emergency assistance, thereby enhancing their self-management capabilities and sense of security [57]. For patients unable to access digital technologies, effective transitional care should be provided, combining psychological support, personalized education, integrated community services, and flexible technological approaches tailored to individual capabilities and preferences [58].

## Limitations

To our knowledge, research focusing on the psychological and behavioral aspects of the OHELP during the transition period following hospital discharge was limited. The findings of this study offered valuable insights for self-management strategies tailored to the OHELP, thereby contributing to the enhancement of their quality of life. Nonetheless, several limitations of this study warrant acknowledgment. Firstly, despite the researchers’ expertise in qualitative research, the findings remained susceptible to subjective interpretation, introducing a certain degree of bias. Secondly, this study primarily focused on the pre-discharge period and did not explore the long-term dynamic changes in psychological and behavioral experiences post-discharge. Future longitudinal studies should track patients’ experiences over an extended period to identify potential shifts in needs and adaptation strategies. Thirdly, due to factors such as willingness to participate, there was a gender imbalance among the participants. This imbalance may have limited the study’s ability to thoroughly explore potential gender differences in the psychological experiences of older adults with heart failure during the discharge preparation period. Future studies should aim for more balanced samples to systematically examine gender differences. Lastly, the perspectives of healthcare providers and family members were not included, which might restrict a comprehensive understanding of support system gaps. Future mixed-methods research could integrate qualitative and quantitative approaches, incorporating perspectives from multiple stakeholders to develop more comprehensive intervention strategies.

## Conclusion

This study revealed that the OHELP experienced significant anticipatory psychological burden during discharge preparation, including fear of medical crises, anticipated loneliness, and vigilance fatigue. A critical mismatch existed between their prioritized support needs—practical guidance for solitary living, clear service pathways, and care continuity—and actual resources. In response, patients developed adaptive strategies including daily routine reestablishment, experiential knowledge construction, strategic dependence, and technological literacy, while actively reconstructing self-worth through meaning-making. These findings highlighted that this population demonstrated remarkable agency despite vulnerabilities. Transitional care should move beyond standardized discharge instructions to provide psychologically attuned, personalized support that integrates community resources and flexible technology approaches tailored to individual capabilities, ensuring a safe and dignified hospital-to-home transition.

## Data Availability

Information regarding patient interviews collected during this study is not publicly available, however, it can be obtained from the corresponding author upon reasonable request.

## Abbreviations

CSM: Common-Sense Model of Self-Regulation
OHELP: Older Heart Failure Patients Living Alone

## References

[1] National Bureau of Statistics. Wang Pingping: The total population decreased slightly and the urbanization level continued to improve. Available from: http://www.stats.gov.cn/xxgk/jd/sjjd2020/202301/t20230118_1892285.html. Accessed 12 December 2025.

[2] Central Government of the People’s Republic of China. Notice of the state council on the issuance of the 14th five-year plan for the development of the national cause for the aged and the planning of the service system for the aged. Available from: https://www.gov.cn/zhengce/zhengceku/2022-03/01/content_5676342.htm. Accessed 12 December 2025.

[3] Ponikowski P, Voors AA, Anker SD, et al. 2016 ESC Guidelines for the diagnosis and treatment of acute and chronic heart failure: The Task Force for the diagnosis and treatment of acute and chronic heart failure of the European Society of Cardiology (ESC)Developed with the special contribution of the Heart Failure Association (HFA) of the ESC. Eur Heart J. 2016;37(27):2129–200. 10.1093/eurheartj/ehw128. 27206819 10.1093/eurheartj/ehw128

[4] Markle-Reid M, Fisher K, Walker KM, et al. The stroke transitional care intervention for older adults with stroke and multimorbidity: a multisite pragmatic randomized controlled trial. BMC Geriatr. 2023;23(1):687. 10.1186/s12877-023-04403-1. 37872479 10.1186/s12877-023-04403-1 PMC10594728

[5] Palonen M, Kaunonen M, Åstedt-Kurki P. Family involvement in emergency department discharge education for older people. J Clin Nurs. 2016;25(21–22):3333–44. 10.1111/jocn.13399. 27218600 10.1111/jocn.13399

[6] Morkisch N, Upegui-Arango LD, Cardona MI, et al. Components of the transitional care model (TCM) to reduce readmission in geriatric patients: a systematic review. BMC Geriatr. 2020;20(1):345. 10.1186/s12877-020-01747-w. 32917145 10.1186/s12877-020-01747-w PMC7488657

[7] Niu XN, Wen H, Sun N, Zhao R, Wang T, Li Y. Exploring risk factors of short-term readmission in heart failure patients: A cohort study. Front Endocrinol (Lausanne). 2022;13:1024759. 10.3389/fendo.2022.1024759. Published 2022 Nov 28. 36518258 10.3389/fendo.2022.1024759 PMC9742544

[8] Graven LJ, Durante A, Abbott L, et al. Self-care Problems and Management Strategies Experienced by Rural Patient/Caregiver Dyads Living With Heart Failure: A Qualitative Study. J Cardiovasc Nurs. 2024;39(3):207–18. 10.1097/JCN.0000000000001056. 37955387 10.1097/JCN.0000000000001056

[9] Chen H, Liu F, Luo J, Tu Y, Huang S, Zhu W. Association of living alone with clinical outcomes in patients with heart failure: A systematic review and meta-analysis. Clin Cardiol. 2024;47(1):e24153. 10.1002/clc.24153. 37740434 10.1002/clc.24153 PMC10765994

[10] Salah HM, Ambrosy AP, Biegus J, et al. Optimizing pre-to-post discharge transition of care in patients hospitalized for heart failure - part 3 of the international expert opinion series on acute heart failure management. J Card Fail Published online September. 2025;28. 10.1016/j.cardfail.2025.09.020. 10.1016/j.cardfail.2025.09.020 41027508

[11] Zhou J, Zhao J, Xin J, Guo Y, Jiang E, Chen C. Experiences of Patients with Heart Failure in Transition from Hospital to Home in China: A Qualitative Study. J Multidiscip Healthc. 2025;18:5505–19. 10.2147/JMDH.S537560. Published 2025 Sep 3. 40923017 10.2147/JMDH.S537560 PMC12414461

[12] Sun M, Qian Y, Liu L, et al. Transition of care from hospital to home for older people with chronic diseases: a qualitative study of older patients’ and health care providers’ perspectives. Front Public Health. 2023;11:1128885. 10.3389/fpubh.2023.1128885. Published 2023 Apr 27. 37181713 10.3389/fpubh.2023.1128885 PMC10174044

[13] Zheng G, Zhou B, Fang Z, et al. Living alone and the risk of depressive symptoms: a cross-sectional and cohort analysis based on the China Health and Retirement Longitudinal Study. BMC Psychiatry. 2023;23(1):853. 10.1186/s12888-023-05370-y. 37978367 10.1186/s12888-023-05370-y PMC10655346

[14] Suksatan W, Tankumpuan T, Davidson PM. Heart Failure Caregiver Burden and Outcomes: A Systematic Review. J Prim Care Community Health. 2022;13:21501319221112584. 10.1177/21501319221112584. 35938489 10.1177/21501319221112584 PMC9364181

[15] Mowery BD, Brand E, Gisila D, et al. Improving Discharge Education and Outcomes for Patients with Heart Failure. Am J Nurs. 2025;125(3):40–6. 10.1097/AJN.0000000000000030. 39972586 10.1097/AJN.0000000000000030

[16] Wiśnicka A, Lomper K, Uchmanowicz I. Self-care and quality of life among men with chronic heart failure. Front Public Health. 2022;10:942305. 10.3389/fpubh.2022.942305. 35937256 10.3389/fpubh.2022.942305 PMC9354614

[17] Tian L, Tian Y, Sun P, et al. Social support and resilience as mediating factors in the link between illness perception and fear of progression among older patients with chronic heart failure: A multiple mediator model. Geriatr Nurs. 2025;65:103521. 40639078 10.1016/j.gerinurse.2025.103521

[18] Shimode Y, Kitai T, Iwata K, et al. Impact of stress coping style on self-care behaviors and prognosis in patients with heart failure: A prospective longitudinal observational study. Int J Cardiol. 2025;421:132865. 10.1016/j.ijcard.2024.132865. 39622346 10.1016/j.ijcard.2024.132865

[19] Leventhal H, Phillips LA, Burns E. The Common-Sense Model of Self-Regulation (CSM): a dynamic framework for understanding illness self-management. J Behav Med. 2016;39(6):935–46. 10.1007/s10865-016-9782-2. 27515801 10.1007/s10865-016-9782-2

[20] Koontalay A, Botti M, Hutchinson A. Illness perceptions of people living with chronic heart failure and limited community disease management. J Clin Nurs. 2024;33(10):4100–11. 10.1111/jocn.17335. 38923175 10.1111/jocn.17335

[21] Yang H, Tian J, Li J, et al. Social and therapeutic decline earlier than physical and psychological domains after discharge in heart failure patients: A patient-reported outcome measurements of latent transition analysis. Front Cardiovasc Med. 2022;9:965201. 10.3389/fcvm.2022.965201. 36204569 10.3389/fcvm.2022.965201 PMC9530707

[22] Younas A, Pedersen M, Tayaben JL. Review of Mixed-Methods Research in Nursing. Nurs Res. 2019;68(6):464–72. 10.1097/NNR.0000000000000372. 31693552 10.1097/NNR.0000000000000372

[23] Li Y, Li C, Zhou N, et al. Perspectives of nurses and patients on the misplacement of supportive care information for type 2 diabetes mellitus: a qualitative study in China. BMC Nurs. 2025;24(1):123. 10.1186/s12912-025-02783-w. 39901206 10.1186/s12912-025-02783-w PMC11792742

[24] Chen C, Qian Y, Wan G, Zhang Z. Exploring Health Literacy Needs in Different Stages of Older Patients with COPD: An in-Depth Qualitative Study. Patient Prefer Adherence. 2025;19:3421–33. 10.2147/PPA.S550268. 41216211 10.2147/PPA.S550268 PMC12596832

[25] Tong A, Sainsbury P, Craig J. Consolidated criteria for reporting qualitative research (COREQ): a 32-item checklist for interviews and focus groups. Int J Qual Health Care. 2007;19(6):349–57. 10.1093/intqhc/mzm042. 17872937 10.1093/intqhc/mzm042

[26] Ni Z, Zhu X, Guo J, Xie S, Liu S, Yang X. Preferences for home-based care services during China’s long-term care market transition: evidence from a discrete choice experiment. BMC Health Serv Res. 2025;25(1):713. Published 2025 May 17. 10.1186/s12913-025-12853-z. 10.1186/s12913-025-12853-z PMC12084924 40380142

[27] Liljeroos M, Andreae C, Jaarsma PT, Wennerholm C. Older heart failure patients’ experiences of follow-up in primary care after discharge from hospital. Geriatr Nurs. 2024;59:458–62. 10.1016/j.gerinurse.2024.07.036. 39141953 10.1016/j.gerinurse.2024.07.036

[28] Malterud K, Siersma VD, Guassora AD. Sample Size in Qualitative Interview Studies: Guided by Information Power. Qual Health Res. 2016;26(13):1753–60. 10.1177/1049732315617444. 26613970 10.1177/1049732315617444

[29] Braun V, Clarke V. Using thematic analysis in psychology. Qual Res Psychol. 2006;3:77–101.

[30] Dabkowski E, Porter JE, Barbagallo M. A thematic analysis of the perceptions of a community-based exercise program on the health and well-being of ageing adults. Health Soc Care Community. 2021;29(6):1990–7. 10.1111/hsc.13343. 33730401 10.1111/hsc.13343

[31] Postolica R, Iorga M, Savin M, Azoicai D, Enea V. The utility of Leventhal’s model in the analysis of the psycho-behavioral implications of familial cancer - a literature review. Arch Med Sci. 2018;14(5):1144–54. 10.5114/aoms.2016.63149. 30154899 10.5114/aoms.2016.63149 PMC6111358

[32] Chen C, Sun X, Zhang Y, Xie H, Kou J, Zhang H. Fear of progression and quality of life in patients with heart failure: a cross-sectional study on the multiple mediation of psychological distress and resilience. BMC Nurs. 2025;24(1):60. 10.1186/s12912-025-02688-8. Published 2025 Jan 17. 39825270 10.1186/s12912-025-02688-8 PMC11742502

[33] Nordfonn OK, Morken IM, Bru LE, Husebø AML. Patients’ experience with heart failure treatment and self-care-A qualitative study exploring the burden of treatment. J Clin Nurs. 2019;28(9–10):1782–93. 10.1111/jocn.14799. 30667120 10.1111/jocn.14799

[34] Friedmann E, Son H, Thomas SA, Chapa DW, Lee HJ. Sudden Cardiac Death in Heart Failure Trial (SCD-HeFT) Investigators. Poor social support is associated with increases in depression but not anxiety over 2 years in heart failure outpatients. J Cardiovasc Nurs. 2014;29(1):20–8. 10.1097/JCN.0b013e318276fa07. 23321780 10.1097/JCN.0b013e318276fa07 PMC3633678

[35] Yan J, Tian J, Yang H, et al. The Causal Effects of Anxiety-Mediated Social Support on Death in Patients with Chronic Heart Failure: A Multicenter Cohort Study. Psychol Res Behav Manag. 2022;15:3287–96. 10.2147/PRBM.S387222. 36387039 10.2147/PRBM.S387222 PMC9653046

[36] Li X, Luantong J, Jing L, Xingyu Z. Pi Zhognling. Does filial piety bring more happiness to older adults? The Role of Filial Piety Expectation. Acta Psychologica Sinica. 2022,54(11):1381–1390. (Chinese). 10.3724/SP.J.1041.2022.01381.

[37] Hou Q, Zhao Y, Wu Y. Medication adherence trajectories and clinical outcomes in patients with cardiovascular disease: a systematic review and meta-analysis. J Glob Health. 2025;15:04145. 10.7189/jogh.15.04145. 40338356 10.7189/jogh.15.04145 PMC12061002

[38] Communist Party of China Central Committee, State Council. Opinions of the CPC Central Committee and the State Council on Deepening the Reform and Development of Elderly Care Services [Internet]. Beijing: Lexiscn. 2024 Dec 30 [cited 2026 Mar 9]. Available from: https://www.lexiscn.com/law/law-english-1-5610431-T.html.

[39] Oh S, Choi H, Oh EG, Lee JY. Effectiveness of discharge education using teach-back method on readmission among heart failure patients: A systematic review and meta-analysis. Patient Educ Couns. 2023;107:107559. 10.1016/j.pec.2022.11.001. 36411152 10.1016/j.pec.2022.11.001

[40] Squires SS, Kipps AK, Gamuciello S, et al. Improving Discharge Medication Education: Consideration of Health Literacy and Language. Hosp Pediatr. 2025;15(8):e350–9. 10.1542/hpeds.2024-008092. 40623705 10.1542/hpeds.2024-008092

[41] Guo P, East L, Arthur A. Thinking outside the black box: the importance of context in understanding the impact of a preoperative education nursing intervention among Chinese cardiac patients. Patient Educ Couns. 2014;95(3):365–70. 10.1016/j.pec.2014.03.001. 24666774 10.1016/j.pec.2014.03.001

[42] Liu X, Yu HJ, Zhang MZ, et al. The transition of eldercare responsibility and traditional filial piety concepts and its urban-rural differences in China: an age-period-cohort analysis from 2006 to 2017. BMC Public Health. 2024;24(1):1669. 10.1186/s12889-024-19175-5. 38909187 10.1186/s12889-024-19175-5 PMC11193898

[43] Wang LY, Hu ZY, Chen HX, Zhou CF, Tang ML, Hu XY. Differences in regional distribution and inequality in health workforce allocation in hospitals and primary health centers in China: A longitudinal study. Int J Nurs Stud. 2024;157:104816. 10.1016/j.ijnurstu.2024.104816. 38824719 10.1016/j.ijnurstu.2024.104816

[44] Ganz FD, Roeh K, Eid M, et al. The need for palliative and support care services for heart failure patients in the community. Eur J Cardiovasc Nurs. 2021;20(2):138–46. 10.1177/1474515120951970. 33611419 10.1177/1474515120951970

[45] Jiang Y, Shorey S, Nguyen HD, et al. The development and pilot study of a nurse-led HOMe-based HEart failure self-Management Programme (the HOM-HEMP) for patients with chronic heart failure, following Medical Research Council guidelines. Eur J Cardiovasc Nurs. 2020;19(3):212–22. 10.1177/1474515119872853. 31486332 10.1177/1474515119872853

[46] Zhou J, Zhao J, Xin J, et al. Experiences of Patients with Heart Failure in Transition from Hospital to Home in China: A Qualitative Study. J Multidiscip Healthc. 2025;18:5505–19. 10.2147/JMDH.S537560. 40923017 10.2147/JMDH.S537560 PMC12414461

[47] Harkness K, Spaling MA, Currie K, et al. A systematic review of patient heart failure self-care strategies. J Cardiovasc Nurs. 2015;30(2):121–35. 10.1097/JCN.0000000000000118. 24651683 10.1097/JCN.0000000000000118

[48] Liu D, Gao Y, Su X. Self-care ability and associated factors in community-dwelling older adults living with oral frailty using the COM-B model. J Oral Rehabil. 2024;51(8):1530–41. 10.1111/joor.13713. 38725254 10.1111/joor.13713

[49] Chin WY, Wong CKH, Ng CCW, et al. Cultural adaptation and psychometric properties of the Chinese Burden of Treatment Questionnaire (C-TBQ) in primary care patients with multi-morbidity. Fam Pract. 2019;36(5):657–65. 10.1093/fampra/cmz008. 30820558 10.1093/fampra/cmz008

[50] Scherrenberg M, Leenen JP, van der Velde AE, et al. Bringing the hospital to home: Patient-reported outcome measures of a digital health-supported home hospitalisation platform to support hospital care at home for heart failure patients. Digit Health. 2023;9:20552076231152178. 10.1177/20552076231152178. 36762022 10.1177/20552076231152178 PMC9903014

[51] McBeath KCC, Angermann CE, Cowie MR. Digital Technologies to Support Better Outcome and Experience of Care in Patients with Heart Failure. Curr Heart Fail Rep. 2022;19(3):75–108. 10.1007/s11897-022-00548-z. 35486314 10.1007/s11897-022-00548-z PMC9051015

[52] Albulushi A, Al Kindi DI, Moawwad N, et al. Digital health technologies in enhancing patient and caregiver engagement in heart failure management: Opportunities and challenges. Int J Cardiol. 2024;408:132116. 10.1016/j.ijcard.2024.132116. 38703898 10.1016/j.ijcard.2024.132116

[53] Kallas D, Sandhu N, Gandilo C, et al. Use of Digital Health Technology in Heart Failure and Diabetes: a Scoping Review. J Cardiovasc Transl Res. 2023;16(3):526–40. 10.1007/s12265-022-10273-6. 35639339 10.1007/s12265-022-10273-6 PMC9153219

[54] Wang Z, Tocchi C, Chyun D, Kim K, Cong X, Starkweather A. The association between psychological factors and self-care in patients with heart failure: an integrative review. Eur J Cardiovasc Nurs. 2023;22(6):553–61. 10.1093/eurjcn/zvac106. 36351041 10.1093/eurjcn/zvac106

[55] Chronic heart failure. in adults: diagnosis and management. London: National Institute for Health and Care Excellence (NICE); September 3, 2025. 41284793

[56] Ramli DB, Shahar S, Mat S, Ibrahim N, Tohit NM. The effectiveness of preventive home visits on resilience and health-related outcomes among community dwelling older adults: A systematic review. PLoS ONE. 2024;19(7):e0306188. 10.1371/journal.pone.0306188. Published 2024 Jul 1. 38950029 10.1371/journal.pone.0306188 PMC11216600

[57] Saravanan A, Kitner B, Sterner EA, et al. Smart tablet education for healthy living (STEHL) intervention improves digital skills: A usability and acceptability study. Geriatr Nurs. 2025;66(Pt B):103693. 10.1016/j.gerinurse.2025.103693. 41202554 10.1016/j.gerinurse.2025.103693

[58] Kwan AST, Teo KW, Sayampanathan AA, et al. Community care at scale: Singapore’s Active Ageing Centres 2.0 as a model for healthy ageing. Ann Acad Med Singap. 2025;54(12):779–85. 10.47102/annals-acadmedsg.2025239. Published 2025 Dec 9. 41492885 10.47102/annals-acadmedsg.2025239

